# Automated methods for surveillance of surgical site infections.

**DOI:** 10.3201/eid0702.010212

**Published:** 2001

**Authors:** R Platt, D S Yokoe, K E Sands

**Affiliations:** Harvard Medical School and Harvard Pilgrim Health Care, Boston, Massachusetts, USA. richard.platt@channing.harvard.edu

## Abstract

Automated data, especially from pharmacy and administrative claims, are available for much of the U.S. population and might substantially improve both inpatient and postdischarge surveillance for surgical site infections complicating selected procedures, while reducing the resources required. Potential improvements include better sensitivity, less susceptibility to interobserver variation, more uniform availability of data, more precise estimates of infection rates, and better adjustment for patients' coexisting illness.


### Automated Methods for Surveillance of Surgical Site Infections

**Richard Platt,*† Deborah S. Yokoe,† Kenneth E. Sands,‡ and the CDC Eastern Massachusetts Prevention Epicenter Investigators[1]**

*Harvard Medical School and Harvard Pilgrim Health Care, Boston, Massachusetts, USA; †Harvard Medical School and Brigham and Women's Hospital, Boston, Massachusetts, USA; and ‡Harvard Medical School, Beth Israel Deaconess Medical Center, Boston, Massachusetts, USA

Automated data, especially from pharmacy and administrative claims, are available for much of the U.S. population and might substantially improve both inpatient and postdischarge surveillance for surgical site infections complicating selected procedures, while reducing the resources required. Potential improvements include better sensitivity, less susceptibility to interobserver variation, more uniform availability of data, more precise estimates of infection rates, and better adjustment for patients' coexisting illness.

The Centers for Disease Control and Prevention (CDC) recommends routine surveillance for surgical site infections (1); accrediting agencies such as the Joint Commission for Accreditation of Healthcare Organizations require it. Surveillance identifies clusters of infection, establishes baseline risks for infection, provides comparisons between institutions or surgical specialties, identifies risk factors, and permits evaluation of control measures (2). Achieving these goals requires health-care systems to have access to different information types (Table 1).

An ideal surveillance system should have several attributes, including meaningful definitions of infection, consistent interpretation of classification criteria, applicability to procedures performed in both inpatient and ambulatory facilities, ability to detect events after discharge, sufficient precision to distinguish small absolute differences in attack rates, ability to adjust for different distribution of severity of illness across populations, and reasonable cost. Most current systems lack at least one of these attributes; for example, the system recommended by CDC's Hospital Infection Control Practices Advisory Committee (HICPAC) (3) is excellent for clinical decision-making, but some elements are difficult to apply for surveillance purposes. Information required to apply some of its criteria may not be available for all cases; for example, the criterion of recovery of microbial growth from a normally sterile site may be affected by variation in obtaining specimens for culture. Some elements of CDC's National Nosocomial Infections (NNIS) System definition require substantial judgment or interpretation. An example is determining whether purulent drainage is present: An attending physician's diagnosis is sufficient, although the way physicians record or confirm their diagnoses may differ.

Address for correspondence: Richard Platt, 126 Brookline Ave., Suite 200, Boston, MA 02215, USA; fax: 617-859-8112; e-mail: richard.platt@channing.harvard.edu

Table 1. Goals and needs of surgical site infection surveillance (2)

| Goal | Principal needs |
|---|---|
| Control of clusters | |
| Identify clusters of infection. | Real-time detection of events. Attack rates and case-mix adjustment are not a high priority. Should include all patients. |
| Support of quality improvement programs | |
| Establish baseline infection rates. | Sufficient precision to identify absolute differences of a few percent. Typically includes all patients. |
| Comparison of institutions or surgical specialities. | Case-mix-adjusted attack rates. Identical detection methods that are applied and interpreted identically across sites. Sufficient precision. |
| Evaluate control measures (in the usual situation no randomized trial). | Comparably ascertained rates of over time. |
| Research on epidemiology of infection | |
| Identify risk factors. | Detailed data on many attributes of patients and procedures. Population can be small, but must be representative. |

For these reasons, case ascertainment is affected by considerable interobserver variability (4).

Although most surgical site infections become manifest after the patient is discharged from the hospital (5-12), there is no accepted method for detecting them (13). The most widely described method of conducting postdischarge surveillance is questionnaire reporting by surgeons. This method has been shown to have poor sensitivity (15%) and

[1]The CDC Eastern Massachusetts Prevention Epicenter includes Blue Cross and Blue Shield of Massachusetts, CareGroup, Children's Hospital, Harvard Pilgrim Health Care, Partners Healthcare System, Tufts Health Plan, and Harvard Medical School. Investigators include L. Higgins, J. Mason, E. Mounib, C. Singleton, K. Sands, K. Kaye, S. Brodie, E. Perencevich, J. Tully, L. Baldini, R. Kalaidjian, K. Dirosario, J. Alexander, D. Hylander, A. Kopec, J. Eyre-Kelley, D. Goldmann, S. Brodie, C. Huskins, D. Hooper, C. Hopkins, M. Greenbaum, M. Lew, K. McGowan, G. Zanetti, A. Sinha, S. Fontecchio, R. Giardina, S. Marino, J. Sniffen, E. Tamplin, P. Bayne, T. Lemon, D. Ford, V. Morrison, D. Morton, J. Livingston, P. Pettus, R. Lee, C. Christiansen, K. Kleinman, E. Cain, R. Dokholyan, K. Thompson, C. Canning, D. Lancaster.

positive predictive value (28%), even when surgeons are compliant in returning the questionnaires (5). Moreover, a questionnaire-based surveillance system requires substantial resources. Reporting by patients via questionnaires also has poor sensitivity (28%) because many patients do not return questionnaires mailed to them a month after surgery. Telephone questionnaires have been used effectively but are too resource intensive for routine use.

Many procedures must be monitored to allow confident conclusions that relatively small differences in observed attack rates do not reflect chance variations. Identifying these small differences, understanding their cause, and undertaking quality improvement programs to reduce their occurrence would have large consequences when applied to the >45 million surgical procedures performed annually in the United States (14). Reducing the overall infection rate by a quarter of a percent would prevent >100,000 infections per year. For coronary artery bypass surgery alone, a one percentage point decrease in the risk for infection would prevent >3,500 infections per year in the United States (15). Because of the need to observe large numbers of procedures, conducting surveillance for the entire surgical population is desirable. However, to conserve scarce resources, some programs survey only a fraction of their procedures or rotate surveillance among different procedure types.

Determining whether relatively small differences in infection rates result from differences in care rather than in patients' susceptibility to infection requires robust risk-adjustment methods that can take into account different case-mixes in different institutions. Available methods do not have optimal resolution and depend in part on the Anesthesia Society of America (ASA) score (3,16). The ASA score, a subjective assessment of the patient's overall health status, may reflect interobserver variability (17) that can adversely affect stratification of risk for surgical infection (18).

Automated methods to augment current surveillance methods should improve the quality of surveillance for surgical site infections and reduce the resources required. To achieve these goals, surveillance should be based on the growing body of data that health-care systems, including hospitals, physicians' offices, health maintenance organizations (HMOs), and insurance companies, routinely collect during care delivery. Many types of automated data are now or will soon become widely available, including information about patients, surgical procedures, and patients' postoperative courses (Table 2). Three ways to use these data to support surveillance programs are inpatient surveillance, postdischarge surveillance, and case-mix adjustment.

Table 2. Automated health-care data potentially useful for surgical site infection surveillance

| | Availability of this information in specific locations | | |
|---|---|---|---|
| Type of information | Hospitals[a] | Automated medical records in physicians' offices | Payors (HMOs, insurers) |
| Demographic/ personal information | | | |
| Sex | Usually | Usually | Usually |
| Age | Usually | Usually | Usually |
| Smoking status | Rarely | Sometimes | Rarely |
| Body mass index | Rarely | Sometimes | Rarely |
| Preoperative health status | | | |
| Diagnoses | Sometimes | Usually | Usually |
| Procedures | Rarely | Sometimes | Usually |
| Drug therapy | Sometimes | Sometimes | Usually |
| ASA score | Sometimes | Rarely | Rarely |
| Procedure data | | | |
| Type (ICD-9, CPT) | Usually | Sometimes | Usually |
| Duration | Sometimes | Rarely | Rarely |
| Inpatient postoperative care | | | |
| Diagnoses | Usually | Sometimes | Usually |
| Reoperation | Usually | Rarely | Usually |
| Incision and drainage | Usually | Rarely | Sometimes |
| Microbiology data | Usually | Rarely | Rarely |
| Antibiotic therapy | Usually | Rarely | Rarely |
| Postdischarge care | | | |
| Diagnoses | Rarely | Usually | Usually |
| Reoperation in another hospital | Rarely | Sometimes | Usually |
| Incision and drainage | Rarely | Usually | Usually |
| Microbiology data | Rarely | Usually | Sometimes |
| Antibiotic therapy | Rarely | Sometimes | Usually |

[a]Excludes hospital-based physicians' offices.

### Inpatient Surveillance for Surgical Site Infections

One of the most widely available types of automated data useful for inpatient surveillance is antibiotic exposure data from pharmacy dispensing records. Studies have indicated that antibiotic exposure is a sensitive indicator of infection (19,20), since relatively few serious infections are managed without antibiotics. Poor specificity (too many false positives) has been a major problem, however, because antibiotics are so widely used after surgery for extended prophylaxis, empiric therapy of suspected infection, and treatment of infections other than surgical site infections.

One way to improve the usefulness of postoperative antibiotic exposure as a marker of infection is to consider the timing and duration of administration, rather than just its occurrence. Quantitative antibiotic exposure is a measure that reduces the number of false positives by excluding patients who receive a brief course; however, there is a trade-off between sensitivity and specificity. Constructing receiver-operating characteristic curves helps to identify the amount of treatment with the best combination of sensitivity and specificity. For example, acceptable identification of infections after cesarean section was achieved by requiring a criterion of at least 2 days of parenteral antibiotic administration (21). In that study, the sensitivity was 81% and the specificity was 95% compared with infections identified by NNIS surveillance.

Quantitative inpatient antibiotic exposure is useful for identifying infections in coronary artery bypass surgery patients (22). Receiver-operating characteristic curves were used to demonstrate that patients with infections were best identified as those who received postoperative antibiotics for at least 9 days, excluding the first postoperative day. This criterion included both oral and parenteral antibiotics and ignored gaps in administration. This approach has two important implications for surveillance systems: It allows this mechanism to identify patients readmitted for treatment of infection within 30 days of surgery, and automated programs to identify patients who meet this threshold are substantially easier to implement. The 9-day exposure cutoff resulted in greater sensitivity (approximately 90%) for identifying surgical site infections than conventional prospective surveillance (approximately 60%) conducted in the same hospitals. A disadvantage of the antibiotic threshold criterion is that it identifies events that are not surgical site infections, including problematic wounds that do not meet the HICPAC criteria for infection, other types of hospital infections, and other long durations of antibiotic use.

Studies under way will determine the utility of this approach in a larger number of hospitals. Preliminary data from nine hospitals suggest that surveillance for antibiotic use provides useful information. For cesarean section, prospective comparison of a quantitative antibiotic exposure threshold to conventional prospective NNIS surveillance and International Classification of Diseases, 9th Revision (ICD-9), discharge diagnosis codes indicates that antibiotic surveillance has considerably better sensitivity (89%) than either NNIS surveillance (32%) or coded discharge diagnoses (47%). This difference was consistent across hospitals (23).

Quantitative thresholds for antibiotic exposure should be chosen individually for specific surgical procedures, since the value for cesarean section (2 days) differs from that for coronary artery bypass grafting (9 days) and there may be no useful threshold for some procedures. These values may also need to be reassessed as medical practice evolves. It will be important to understand the discrepancies between the results of formal NNIS surveillance and antibiotic surveillance. In some cases, patients who receive more than the threshold duration of antibiotic therapy appear to have clinically relevant infectious illness, such as fever and incisional cellulitis with no drainage.

### Postdischarge Surveillance for Surgical Site Infection

Because most infections become manifest after discharge and many patients with infections never return to the hospital where the surgery was performed (5), traditional inpatient surveillance methods are not sufficient. In addition, conventional methods for postdischarge surveillance, including surgeon questionnaires, are highly inaccurate, with both low sensitivity and specificity.

Information about postdischarge care is available in office-based electronic medical records of coded diagnoses, procedures, tests, and treatments from the automated billing and pharmacy dispensing data maintained by most HMOs and many insurers. Pharmacy dispensing information is typically available for insured patients who have a pharmacy benefit. Together, these automated data elements identified >99% of postdischarge infections that occurred after a mixed group of nonobstetric surgical procedures (5). This high sensitivity came at the cost of low specificity (many false positives requiring manual review of medical records).

Recursive partitioning, logistic regression modeling, and bootstrap methods have made it possible to preserve good sensitivity while improving specificity by combining automated data from inpatient and ambulatory sources. The resulting algorithms use these automated data to assign to each patient an estimated probability for postoperative infection. These probabilities of infection, based on postoperative events that indicate infection has occurred, must be distinguished from predictions based on personal risk factors such as diabetes or obesity or on characteristics of the procedures such as the duration of surgery.

Choosing a lower probability threshold results in higher sensitivity and lower specificity, whereas a higher threshold improves specificity at the expense of sensitivity. For example, using automated data from both HMOs and ambulatory medical records permitted a sensitivity of 74% and a specificity of 98%, for a predictive value positive of 48%. A higher sensitivity, 92%, was achieved at the expense of lowering the specificity to 92%, for a predictive value positive of 21% (Figure) (24).

This work has been extended to surveillance for inpatient and postdischarge surgical site infections following coronary artery bypass surgery in five hospitals (25). That study found that HMO data alone identified 73% of 168 infections and hospital data alone identified 49% of the same infections. Separate algorithms have been developed to identify postpartum infections occurring after discharge (26).

The utility of automated data sources might be improved in several ways: 1) A procedure-specific algorithm will likely perform better than a general one. 2) Algorithms can be improved to further reduce the number of false positives (e.g., by excluding codes for infection that occur on the same day as a surgical procedure or for antibiotics dispensed before the second postoperative day). 3) These algorithms should be made robust enough for general use by including all ICD-9 and Current Procedural Terminology codes that might be used for surgical site infections.

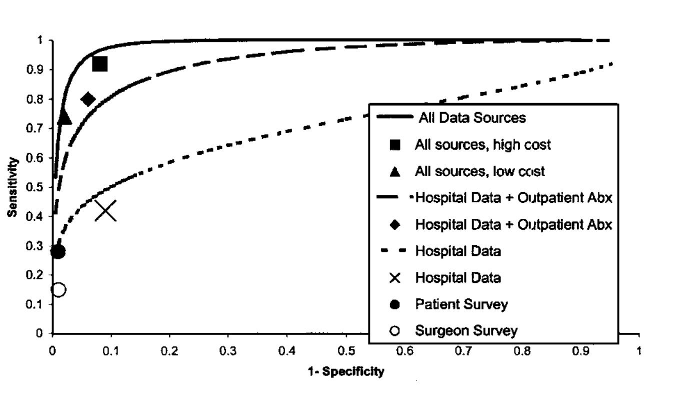

Figure. Performance of various methods for detection of postdischarge surgical site infections for 4,086 nonobstetric surgical procedures with no inpatient infection. Lines represent fitted receiver operating characteristic (ROC) curves for three logistic regression models, which differ by data sources available for generating probabilities. Points represent performance of four different recursive partitioning models and data from patient and physician surveys. For analyses limited to hospital data and outpatient antibiotic (Abx) dispensing data, the logistic regression model had equivalent performance to classification trees at the points shown. The fitted ROC curve falls below this point because most procedures clustered around a few discrete probabilities and limited data points cause approximation of the ROC curve to be less accurate. The recursive partitioning high-cost model accepts 15 false-positives at the margin to capture one true infection; the low-cost model accepts 5 false positives at the margin (24). (Figure originally published in Sands et al. Journal of Infectious Diseases 1999;179:434. Copyright 1999, University of Chicago Press. Reprinted with permission.)

### Improved Case-Mix Adjustment Methods

As quality improvement and patient safety programs evolve, there are likely to be many more opportunities and incentives for comparing infection rates within and across institutions. However, such comparisons will require case-mix adjustment that accounts for coexisting illnesses, to avoid penalizing hospitals that care for patients at higher risk. As discussed, the NNIS risk index is based on the ASA score, which has several undesirable features. Although the ASA score has five possible values, the NNIS index collapses them into two levels so that all information about coexisting illness is summarized, in effect, as high or low. There is often little heterogeneity of ASA score in patients within a surgical procedure class, for instance, cesarean sections. In addition, the ASA score is subject to considerable interobserver variation, is not available for many ambulatory procedures, is usually not captured in automated form by hospital databases, and is not available in administrative or claims data systems.

As an alternative to the ASA score, the chronic disease score has been proposed to adjust data for coexisting illness in surgical patients. This score is based on the premise that dispensed drugs are markers for chronic coexisting illness; for example, dispensing of hypoglycemic agents strongly suggests the presence of diabetes. Approximately 24 conditions are represented in the chronic disease score, which is computed from ambulatory pharmacy dispensing information and can predict death and overall resource use (27-30). The chronic disease score has theoretical advantages over the ASA score: it can be computed automatically for the approximately 90% of the population that has prescription drug coverage, and it is completely objective. In its first application to a mixed group of surgical procedures, the chronic disease score performed at least as well as the ASA score (30). In addition, a modified chronic disease score, based on data for drugs dispensed on hospital admission, performed with substantially better sensitivity and specificity than the ASA score. The chronic disease score, based on admission medications, can also be computed by health-care facilities without the need for ambulatory drug-dispensing data.

The chronic disease score might be considered as a substitute when the ASA score is not available or as a supplement to the ASA score to provide better risk stratification. In addition, the chronic disease score might be modified to optimize its prediction of surgical site infections, rather than all causes of death and resource utilization. For example, data on psychotropic drugs, which are important contributors to the overall chronic disease score, might detract from the prediction of infection. Improved scoring systems will need to be developed through formal modeling programs applied to large, heterogeneous datasets.

### Potential Uses of Electronic Data for Surgical Site Infection Surveillance

Electronic data have the potential to provide better information about infections while reducing the effort required to conduct surveillance. The outcome measures (e.g., quantitative antibiotic exposure or combinations of coded diagnoses) are meaningful, although they differ from the NNIS definition. The medical profession must decide whether a surveillance definition of surgical site infection might coexist with a clinical definition, with the understanding that the two serve related but different purposes (for example, the surveillance definition for influenza epidemics depends on hospitalizations with a coded diagnosis of pneumonia or influenza rather than virologically confirmed infections or specific clinical signs and symptoms).

Implementation of systems that use these data requires consensus on the part of the medical profession about outcome definitions, surveillance algorithms, and reporting standards. Even if consensus is reached, impediments will remain to the widespread adoption of electronic surveillance systems. The disparity in the electronic systems currently in use is one of these. While more sophisticated systems will permit better surveillance, most of the results described above depend on data elements such as drug dispensing information or financial claims data that are already available or are among the first to become automated. Thus, it will not be necessary to wait for fully automated medical records or more advanced hospital information systems. Although the costs of developing and validating systems based on electronic data are substantial, much of the development can be centralized, and validation need only be conducted in a few sites to establish generalizability. These reporting systems require a moderate investment by hospitals, HMOs, and insurers, most of which is the fixed cost for creating automated reporting functions. While some of this cost can be defrayed through the use of standard, shared computer code, this code usually must be customized to make it compatible with existing automated systems. Organizations that have electronic data typically create similar reports for other purposes and will not need new skills. In addition, the costs of maintaining and using the periodic reports that will constitute a new surveillance system are negligible.

Data sharing between hospitals, HMOs, and insurers is important, since very few single entities possess enough information to implement a self-sufficient surveillance system. Furthermore, in many locales, hospitals contract with several HMOs and insurers. In that case, HMOs and insurers must share information among themselves as well as with the hospitals, since no one hospital is likely to have enough patients to achieve the necessary precision. Data sharing will require development of systems that protect both patients' confidentiality and the organizations' proprietary interests.

If such surveillance becomes widely available, two types of uses might coexist. One would be to improve traditional prospective surveillance; for example, sensitivity of inpatient surveillance could be maintained with greatly reduced effort by restricting traditional (NNIS) review to the <10% of records that meet the quantitative screening criterion for antibiotic exposure. Similarly, for the postdischarge surveillance system, one could review as little as 2% of records (including ambulatory records in physicians' offices) while greatly increasing the sensitivity of detection.

A second way to use these surveillance systems is to apply them to the entire surgical population, including patients or procedures that are not being evaluated because of resource constraints. Tracking the proportion of inpatients who exceed the antibiotic threshold or the number of patients who exceed a prespecified computed probability of surgical site infection after discharge might be sufficient, as long as that proportion is within agreed-upon limits. When the rates are below this limit, no further evaluation would be needed, since important problems in the delivery system are unlikely to have escaped detection. However, when the proportion or number exceeds the prespecified limit, more rigorous examination of the data would be triggered.

Electronically assisted surveillance for infections could be performed at modest expense by many organizations that have administrative claims and pharmacy data. These groups include the providers of care for most of the U.S. population, including essentially all HMO members, many of those with traditional indemnity insurance, Medicaid recipients, and most Medicare beneficiaries who have pharmacy benefits.

Supported in part by cooperative agreement UR8/CCU115079 from CDC.

Dr. Platt is professor of ambulatory care and prevention at Harvard Medical School, hospital epidemiologist at Brigham and Women's Hospital, and director of research at Harvard Pilgrim Health Care, an HMO.

### References

1. Haley RW, Culver DH, White JW, Morgan WM, Emori TG, Munn VP, et al. The efficacy of infection surveillance and control programs in preventing nosocomial infections in US hospitals. Am J Epidemiol 1985;121:182-205.
2. Gaynes RP, Horan TC. Surveillance of nosocomial infections. In: C.G. Mayhall, editor. Hospital epidemiology and infection control. 2nd ed. Baltimore: Lippincott, Williams and Wilkins, 1999. Chapter 85.
3. Mangram AJ, Horan TC, Pearson ML, Silver LC, Jarvis WL, Guideline for the prevention of surgical site infection, 1999. Infect Control Hosp Epidemiol 1999;20:247-78.
4. Emori TG, Edwards JR, Culver DH, Sartor C, Stroud LA, Gaunt EE, et al. Accuracy of reporting nosocomial infections in intensive-care-unit patients to the National Nosocomial Infections Surveillance system: a pilot study. Infect Control Hosp Epidemiol 1998;19:308-16.
5. Sands K, Vineyard G, Platt R. Surgical site infections occurring after hospital discharge. J Infect Dis 1996;173:963-70.
6. Reimer K, Gleed G, Nicolle LE. The impact of postdischarge infection on surgical wound infection rates. Infect Control 1987;8:237-40.
7. Manian FA, Meyer L. Comprehensive surveillance of surgical wound infections in outpatient and inpatient surgery. Infect Control Hosp Epidemiol 1990;11:515-20.
8. Burns SJ. Postoperative wound infections detected during hospitalization and after discharge in a community hospital. Am J Infect Control 1982;10:60-5.
9. Polk BF, Shapiro M, Goldstein P, Tager I, Gore-White B, Schoenbaum SC. Randomised clinical trial of perioperative cefazolin in preventing infection after hysterectomy. Lancet 1980;1:437-41.
10. Brown RB, Bradley S, Opitz E, Cipriani D, Pieczrka R, Sands M. Surgical wound infections documented after hospital discharge. Am J Infect Control 1987;15:54-8.
11. Byrne DJ, Lynce W, Napier A, Davey P, Malek M, Cuschieri A. Wound infection rates: the importance of definition and post-discharge wound surveillance. J Hosp Infect 1994;26:37-43.
12. Holtz TH, Wenzel RP. Postdischarge surveillance for nosocomial wound infection: a brief review and commentary. Am J Infect Control 1992;20:206-13.
13. Sherertz RJ, Garibaldi RA, Marosok RD. Consensus paper on the surveillance of surgical site infections. Am J Infect Control 1992;20:263-70.
14. Owings MF, Kozak LJ. Ambulatory and inpatient procedures in the United States, 1996. National Center for Health Statistics. Vital Health Stat 1999;13:139.
15. Lawrence L, Hall MJ. National Center for Health Statistics. 1977 Summary: National Hospital Survey. Advance Data. 1999;308:1-16.
16. Garibaldi RA, Cushing D, Lerer T. Risk factors for postoperative infection. Am J Med 1991;91:158S-163S.
17. Haynes SR, Lawler PG. An assessment of the consistency of ASA physical status classification allocation [see comments]. Anaesthesia 1995;50:195-9.
18. Salemi C, Anderson D, Flores D. American Society of Anesthesiology scoring discrepancies affecting the National Nosocomial Infection Surveillance System: surgical-site-infection risk index rates. Infect Control Hosp Epidemiol 1997;18:246-7.
19. Wenzel R, Osterman C, Hunting K, Galtney J. Hospital-acquired infections. I. Surveillance in a university hospital. Am J Epidemiol 1976;103:251-60.
20. Broderick A, Motomi M, Nettleman M, Streed S, Wenzel R. Nosocomial infections: validation of surveillance and computer modeling to identify patients at risk. Am J Epidemiol 1990;131:734-42.
21. Hirschhorn L, Currier J, Platt R. Electronic surveillance of antibiotic exposure and coded discharge diagnoses as indicators of postoperative infection and other quality assurance measures. Infect Control Hosp Epidemiol 1993;14:21-8.
22. Yokoe DS, Shapiro M, Simchen E, Platt R. Use of antibiotic exposure to detect postoperative infections. Infect Control Hosp Epidemiol 1998;19:317-22.
23. Yokoe DS. Enhanced methods for inpatient surveillance of surgical site infections following cesarean delivery [Abstract S-T3-03]. Fourth Decennial International Conference on Healthcare-Associated and Nosocomial Infections. 2000 Mar 5-9; Atlanta, GA; Centers for Disease Control and Prevention.
24. Sands K, Vineyard G, Livingston J, Christiansen C, Platt R. Efficient identification of postdischarge surgical site infections using automated medical records. J Infect Dis 1999;179:434-41.
25. Sands K, Yokoe D, Hooper D, Tully, Platt R. Multi-institutional comparison of surgical site infection surveillance by screening of administrative and pharmacy data [Abstract M35]. Society of Healthcare Epidemiologists, Annual meeting; Apr 18-20 1999; San Francisco.
26. Yokoe DS, Christiansen C, Sands K, Platt R. Efficient identification of postpartum infections occurring after discharge [Abstract P-T1-20]. 4th Decennial International Conference on Healthcare-associated and Nosocomial Infections. 2000 Mar 5-9; Atlanta, GA. Centers for Disease Control and Prevention.
27. Von Korff M, Wagner EH, Saunders K. A chronic disease score from automated pharmacy data. J Clin Epidemiol 1992;45:197-203.
28. Fishman P, Goodman M, Hornbrook M, Meenan R, Bachman D, O'Keefe-Rosetti M. Risk adjustment using automated pharmacy data: a global Chronic disease score. 2nd International Health Economic Conference, Rotterdam, the Netherlands, 1999.
29. Clark DO, Von Korff M, Saunders K, Baluch WM, Simon GE. A chronic disease score with empirically derived weights. Med Care 1995;33:783-95.
30. Kaye KS, Sands K, Donahue JG, Chan A, Fishman P, Platt R. Preoperative drug dispensing predicts surgical site infection. Emerg Infect Dis 2001;7:57-64.

## References

[PDF_00530] Broderick A., Mori M., Nettleman M. D., Streed S. A., Wenzel R. P. (1990). Nosocomial infections: validation of surveillance and computer modeling to identify patients at risk.. Am J Epidemiol.

[PDF_00501] Brown R. B., Bradley S., Opitz E., Cipriani D., Pieczarka R., Sands M. (1987). Surgical wound infections documented after hospital discharge.. Am J Infect Control.

[PDF_00494] Burns S. J., Dippe S. E. (1982). Postoperative wound infections detected during hospitalization and after discharge in a community hospital.. Am J Infect Control.

[PDF_00504] Byrne D. J., Lynch W., Napier A., Davey P., Malek M., Cuschieri A. (1994). Wound infection rates: the importance of definition and post-discharge wound surveillance.. J Hosp Infect.

[PDF_00565] Clark D. O., Von Korff M., Saunders K., Baluch W. M., Simon G. E. (1995). A chronic disease score with empirically derived weights.. Med Care.

[PDF_00481] Emori T. G., Edwards J. R., Culver D. H., Sartor C., Stroud L. A., Gaunt E. E., Horan T. C., Gaynes R. P. (1998). Accuracy of reporting nosocomial infections in intensive-care-unit patients to the National Nosocomial Infections Surveillance System: a pilot study.. Infect Control Hosp Epidemiol.

[PDF_00518] Garibaldi R. A., Cushing D., Lerer T. (1991). Risk factors for postoperative infection.. Am J Med.

[PDF_00470] Haley R. W., Culver D. H., White J. W., Morgan W. M., Emori T. G., Munn V. P., Hooton T. M. (1985). The efficacy of infection surveillance and control programs in preventing nosocomial infections in US hospitals.. Am J Epidemiol.

[PDF_00520] Haynes S. R., Lawler P. G. (1995). An assessment of the consistency of ASA physical status classification allocation.. Anaesthesia.

[PDF_00534] Hirschhorn L. R., Currier J. S., Platt R. (1993). Electronic surveillance of antibiotic exposure and coded discharge diagnoses as indicators of postoperative infection and other quality assurance measures.. Infect Control Hosp Epidemiol.

[PDF_00507] Holtz T. H., Wenzel R. P. (1992). Postdischarge surveillance for nosocomial wound infection: a brief review and commentary.. Am J Infect Control.

[PDF_00568] Kaye K. S., Sands K., Donahue J. G., Chan K. A., Fishman P., Platt R. (2001). Preoperative drug dispensing as predictor of surgical site infection.. Emerg Infect Dis.

[PDF_00516] Lawrence L., Hall M. J. (1999). 1997 summary: National Hospital Discharge Survey.. Adv Data.

[PDF_00491] Manian F. A., Meyer L. (1990). Comprehensive surveillance of surgical wound infections in outpatient and inpatient surgery.. Infect Control Hosp Epidemiol.

[PDF_00497] Polk B. F., Tager I. B., Shapiro M., Goren-White B., Goldstein P., Schoenbaum S. C. (1980). Randomised clinical trial of perioperative cefazolin in preventing infection after hysterectomy.. Lancet.

[PDF_00488] Reimer K., Gleed C., Nicolle L. E. (1987). The impact of postdischarge infection on surgical wound infection rates.. Infect Control.

[PDF_00523] Salemi C., Anderson D., Flores D. (1997). American Society of Anesthesiology scoring discrepancies affecting the National Nosocomial Infection Surveillance System: surgical-site-infection risk index rates.. Infect Control Hosp Epidemiol.

[PDF_00546] Sands K., Vineyard G., Livingston J., Christiansen C., Platt R. (1999). Efficient identification of postdischarge surgical site infections: use of automated pharmacy dispensing information, administrative data, and medical record information.. J Infect Dis.

[PDF_00486] Sands K., Vineyard G., Platt R. (1996). Surgical site infections occurring after hospital discharge.. J Infect Dis.

[PDF_00559] Von Korff M., Wagner E. H., Saunders K. (1992). A chronic disease score from automated pharmacy data.. J Clin Epidemiol.

[PDF_00527] Wenzel R. P., Osterman C. A., Hunting K. J., Gwaltney J. M. (1976). Hospital-acquired infections. I. Surveillance in a university hospital.. Am J Epidemiol.

[PDF_00538] Yokoe D. S., Shapiro M., Simchen E., Platt R. (1998). Use of antibiotic exposure to detect postoperative infections.. Infect Control Hosp Epidemiol.

